# The Effects of Rope Training on Lymphocyte ABCA1 Expression, Plasma ApoA-I and HDL-c in Boy Adolescents

**DOI:** 10.5812/ijem.8178

**Published:** 2013-04-01

**Authors:** Bahloul Ghorbanian, Aliasghar Ravassi, Mohammad Reza Kordi, Mahdi Hedayati

**Affiliations:** 1 Physical Education and Sports Sciences Department, Azarbaijan Shahid Madani University, Tabriz, IR Iran; 2 Faculty of Physical Education and Sports Sciences, Tehran University, Tehran, IR Iran; 3 Obesity Research Center, Research Institute for Endocrine Sciences, Shahid Beheshti University of Medical Sciences, Tehran, IR Iran

**Keywords:** ABCA1, Apolipoprotein A-I, Rope Training, Overweight and Obese Boy Adolescents

## Abstract

**Background:**

Early obesity and its transfer to the adulthood, increases likelihood incidence of coronary artery disease (CAD). ATP-binding cassette transporter (ABCA1) as a member of the ABC transporters family plays a crucial role in reverse cholesterol transport and CAD prevention.

**Objective:**

The current study aimed to investigate ABCA1 expression in lymphocytes, plasma apolipoprotein A-I and HDL-C in response to eight-week interval endurance rope training in overweight and obese boy adolescents.

**Patients and Methods:**

Thirty students (17.3 ± 1.1 yr, 85.73 ± 11.68 kg and 28.41 ± 2.36 kg / m²) volunteered and were randomly assigned into training (n= 15) and control (n = 15) groups. Exercise protocol was interval endurance rope training (8 wk, 4 d/wk and 40 min/d). Cell hemolysis and sensitive Elisa method was used for Lymphocyte ABAC1 protein expression.t-test was employed.

**Results:**

The independent-samples T-Test results showed that after 8 weeks IERT, the levels of lymphocyte ABCA1 expression (P = 0/001) and VO2max(P = 0/001) significantly increased and plasma levels of TG (P = 0.017), TC (P = 0.001), LDL-c/HDL-c (P = 0.026),TC/HDL-c (P = 0.002) and measures of BF% (P = 0/015) and BMI (P = 0.042) as anthropometric indicators significantly decreased. Changes of other variables such as increase in ApoA-I, HDL-c and decrease in LDL-c, body weight, were not significant.

**Conclusions:**

The findings of this study proved that eight-week interval endurance rope training can have positive effects on lymphocyte ABCA1 protein expression (as gatekeeper of reverse cholesterol process) and lipid profiles among overweight and obese boy adolescents.

## 1. Background

The prevalence of obesity is increasing at an alarming rate (1, 2). This phenomenon extends to children and adolescents in all countries of the industrialized world (1). Iran is also a growing trend in the prevalence of obesity and overweight (3). The association between low levels of high-density lipoprotein cholesterol (HDL-C) and an increased risk for coronary artery disease (CAD) has been well established through epidemiological and clinical studies (4). It is generally accepted that atheroprotective role of HDL is because of its role in reverse cholesterol transport (RCT) (5). Reverse cholesterol transport is a metabolic pathway where by excess cholesterol in peripheral tissues is transported to the liver for elimination from body (6). ABCA1 that is widely expressed in macrophages, liver, small intestine, adrenal glands, endothelial cells and placental trophoblastic (7, 8) and liver producer lipid-poor apolipoproteinA-I, plays an important role in plasma HDL-cholesterol metabolism and reveres cholesterol (9, 10). By a complex series of steps involving acquisition of more lipids and proteins and esterification of cholesterol, this partially lapidated ApoA-I matures into spherical particles that represent the bulk of HDL. These particles are processed and remodeled by the combined actions of cholesteryl ester transfer protein (CETP), phospholipid transfer protein (PLTP), scavenger receptor B1 (SR-B1), and hepatic lipase(HL), which transfer HDL cholesterol esters to other lipoproteins and cells and regenerate lipid-poor ApoA-I. The gatekeeper of this reverse cholesterol transport pathway is an ABCA1 (11, 12). It is believed that an increase in liver and small intestine ApoA-I release and higher ABCA1 expression in macrophage have strong impact on reverse cholesterol transport (RCT) process, plasma HDL-C formation and protection against atherosclerosis (13, 14). Also it has been suggested that ABCA1 is unregulated by several factors such as cholesterol influx, nutritional status, plasma glucose concentrations and physical activity (15, 16). A review of the few existing studies on human samples such Butcher et al. (17), Ghanbari-Niaki et al. (18) and Rashidlamiret al. (19) reflects the positive effect of exercise on ABCA1 expression and improvement of the RCT. Lack of sufficient activity in children and adolescents, and prevalence of early obesity increase likelihood of obesity-related diseases particularly CAD in adulthood, and since the ABCA1 has an important role in RCT and subsequently the prevention of CAD, in the current study, researcher and colleagues intended to explore the effects of eight-week interval aerobic rope training program on lymphocyte ABCA1 protein expression, plasma apolipoprotein A-I and lipid profiles in overweight and obese boy adolescents. It was the first time that young human samples with the characteristics of obese and overweight, were studied under a unique exercise protocol implemented with advantages such as simplicity, low cost and being carried out at intervals which can be considered as important points regarding the current study. According to the American Sports Medicine Research Association, intermittent physical activity has beneficial effects on the cardiovascular system, lipid profiles, blood pressure, insulin resistance and body weight and leading to the remaining people in sports(20, 21).

## 2. Objectives

The current study aimed to investigate ABCA1 expression in lymphocytes, plasma apolipoprotein A-I and HDL-C in response to eight-week interval endurance rope training in overweight and obese boy adolescents.

## 3. Patients and Methods

### 3.1. Participants

Thirty obese and overweight (age 14-17 yr, 85.73± 11.68 kg, 28.41± 2.36 kg / m²) male adolescents were recruited from a high school in Takap City (West Azarbaijan Province, Iran). This study was approved by an institutional ethics review board at the department of sport physiology department of physical education faculty in Tehran University. All subjects provided their written informed consent to participate in the study. Subjects were asked to complete a medical examination and a medical questionnaire to ascertain that they did not take any medication, were free of cardiac, respiratory, renal, and metabolic diseases. Subjects were not participating in any regular physical activities except school physical education class. Body weight was measured with a digital scale (sensitivity of 0.1 kg) and height was measured to the nearest millimeter using JENIX (DS-102, Korea). BMI was calculated as weight in kilograms divided by the square of height in meters. Body fat percentage was calculated by Lipid Caliper with sensitivity of 0.2mm (YAGAMI, Japan) using Jackson and Pollock 3-site skinfold equations (22). Also VO2 max was estimated by one mile Rockport Fitness Walking Test (23).

### 3.2. Study Design

Once subjects were recruited and baseline measurements were completed, they were randomly assigned into exercise (n = 15) and control (n =15) groups. Subjects in the exercise group participated in jump roping exercise in addition to regular physical education class, while the control group participated in only a regular physical education class. Anthropometric variables, lymphocyte ABAC1 protein expression andplasma Apo A-I, triglyceride (TG), total cholesterol (TC), low-density lipoprotein-cholesterol (LDL-C), high-density lipoprotein-cholesterol(HDL-C)and fasting blood sugar were measured before and after 8 weeks of jump rope exercise.

### 3.3. Exercise Training

Subjects in the exercise group participated in supervised interval endurance rope training four times per week, 40 min/d for 8 weeks. The detailed exercise training program is summarized in Table 1.

**Table 1.** Interval Endurance Rope Training Program

Week	Intensity (jumps, min)	Warm-up (5 mins)	Exercise Duration (30 mins)	Cool Down (5 mins)
**1**	60	Stretching	1 min of exercise, 30 secs of rest	Stretching
**2**	60		1.5 min of exercise, 30 secs of rest	
**3**	60		2min of exercise, 30 secs of rest	
**4**	70		2.5 min of exercise, 30 secs of rest	
**5**	80		3 min of exercise, 30 secs of rest	
**6**	90		3.5 min of exercise, 30 secs of rest	
**7**	90		4 min of exercise, 30 secs of rest	
**8**	90		4 min of exercise, 30 secs of rest	

### 3.4. Blood Collection and Lymphocyte Preparation

Participants attended the laboratory 48 hours before the first, and 48 hours after the last sessions, at 8 A.M, after an overnight fasting and after having been abstained from exercise. A 10cc fasting venous blood from brachial vein was obtained. The collected blood samples were collected (3cc for lymphocyte isolation and 7cc for plasma variables measure) in test tubes were anticoagulated with EDTA. Plasma was separated by centrifuging for 15 minutes at 1000 ×g at 2-8°C within 30 minutes of collection and divided into three aliquots. The aliquots were frozen and stored at −80 °C for subsequent analyses (within 2–3 weeks). In order to isolate lymphocytes, 3 cc of EDTA anticoagulated samples were mixed with 5 ml lubricating buffer [preparing by: 50mmol Tris base (PH=7.6), 5mmol Carver magnesium (0.5 mmolar), 109gr sucrose, 5mmolar Triton X (1%) and 1 liter distilled water] and centrifuged at 3000 rpm for 15 minutes and the supernatant was again mixed with 5 ml phosphate buffered saline and centrifuged at 3000 rpm for 15 minutes. Finally the isolated white blood cells were frozen and stored at −80 °C for further experiments.

Cell hemolysis and the sensitive ELISA method were used to measure the lymphocyte ABCA1 protein expression. ELISA assay of this protein on cell lysis had been previously reported (24). Frist, after preparing cell lysates by lysis buffer (Tris 50 mM , 5 Mm EDTA , 1% Triton X-100, pH 7.6) containing complete protease inhibitor (pro-block cocktails anti-protease, manufacturers Gouldbio Inc. USA) which were performed on ice, supernatants containing clear cell lysates were collected after centrifugation at 15,000 rpm for 15 minutes at 4 ° C by refrigerated centrifuge (Hythe, Germany). The clear leukocyte cell lysates were stored at −80°C until ABCA1 protein measurement was performed. Frozen leukocyte lysates were thawed and ABCA1 protein concentration was measured by ELISA method (Human ABCA1 ELISA Kit, Cusabio, china). Very sensitive biotin-streptavidin protocol had been used in the kit. Measurement was performed according to kit instructions.

ApoA-1 was determined by ELISA method (Human Apo A-I, Elisa, Assay proInc, USA). The assay sensitivity of this kit was 1.2 μg/ml and the intra- and inter-assay coefficient of variation was 3.7 and 7.3% respectively. HDL-c and LDL-C were determined by enzymatic colorimetric methods using commercially available kits (Randox, County Antrim, UK). Plasma total Triglyceride (TG) was determined by enzymatic colorimetric method by Glycerol-3-Phosphate Oxidase (GPO) (Pars Azmoun, Tehran, Iran). The intra-assay coefficient of variation and sensitivity of the method were 2.2% and 1 mg/dL respectively. Plasma total cholesterol (TC) was determined by enzymatic photometric method by using Cholesterol Oxidase-Amino Antipyrine(CHOD-PAP) (Pars Azmoun, Tehran, Iran), the intra-assay coefficient of variation and sensitivity of the method were 1.9% and 0.08mmol/L respectively. Fasting plasma glucose (FPG) was measured by an enzymatic colorimetric method using glucose oxidase (Pars azmoun, Tehran, Iran). The intra-assay coefficient of variation and sensitivity of the method were 2.3% and 5 mg/dL respectively.

### 3.5. Leukocyte ABCA1 Protein Expression 

Cell hemolysis and the sensitive ELISA method were used to measure the lymphocyte ABCA1 protein expression. ELISA assay of this protein on cell lysis had been previously reported (24). Frist, after preparing cell lysates by lysis buffer (Tris 50 mM , 5 Mm EDTA , 1% Triton X-100, pH 7.6) containing complete protease inhibitor (pro-block cocktails anti-protease, manufacturers Gouldbio Inc. USA) which were performed on ice, supernatants containing clear cell lysates were collected after centrifugation at 15,000 rpm for 15 minutes at 4 ° C by refrigerated centrifuge (Hythe, Germany). The clear leukocyte cell lysates were stored at −80°C until ABCA1 protein measurement was performed. Frozen leukocyte lysates were thawed and ABCA1 protein concentration was measured by ELISA method (Human ABCA1 ELISA Kit, Cusabio, china). Very sensitive biotin-streptavidin protocol had been used in the kit. Measurement was performed according to kit instructions.

### 3.6. Biochemical Analyses

ApoA-1 was determined by ELISA method (Human Apo A-I, Elisa, Assay proInc, USA). The assay sensitivity of this kit was 1.2 μg/ml and the intra- and inter-assay coefficient of variation was 3.7 and 7.3% respectively. HDL-c and LDL-C were determined by enzymatic colorimetric methods using commercially available kits (Randox, County Antrim, UK). Plasma total Triglyceride (TG) was determined by enzymatic colorimetric method by Glycerol-3-Phosphate Oxidase (GPO) (Pars Azmoun, Tehran, Iran). The intra-assay coefficient of variation and sensitivity of the method were 2.2% and 1 mg/dL respectively. Plasma total cholesterol (TC) was determined by enzymatic photometric method by using Cholesterol Oxidase-Amino Antipyrine(CHOD-PAP) (Pars Azmoun, Tehran, Iran), the intra-assay coefficient of variation and sensitivity of the method were 1.9% and 0.08mmol/L respectively. Fasting plasma glucose (FPG) was measured by an enzymatic colorimetric method using glucose oxidase (Pars azmoun, Tehran, Iran). The intra-assay coefficient of variation and sensitivity of the method were 2.3% and 5 mg/dL respectively.

### 3.7. Statistics

After confirming normal distribution of data with the Kolmogorov - Smirnov (k-s) test, to compare the difference of variables in pre and post-exercise, the independent t-test was used, and P < 0.05 was considered as criterion of statistical significance. SPSS version 16 was employed to perform the statistical analysis.

## 4. Results

The age, body weight, height, BMI, body fat percent, VO2 max , serum levels of fasting glucose, lipid profiles, plasma level of ApoA-I, lymphocyte ABCA1 protein expression concentration of the two groups at pre and post exercise are shown in Table 2. At baseline, there was no significant differences between the two groups in all variables but in post exercise the levels of lymphocyte ABCA1 expression(P =0/001) and VO2max (P =0/001) significantly increased and plasma levels of TG(P =0.017), TC(P =0.001), LDL-c/HDL-c(0.026),TC/HDL-c(P =0.002)and measures of BF% (P =0/015) and BMI (P =0.042) as anthropometric indicators decreased. Changes of other variables such increase in ApoA-I, HDL-c and decrease in LDL-c, and body weight, were not significant. Significant level (P >0.05) was considered (Table 2).

**Table 2.** Pre and Post Exercise ValuesBetween Subjects Of Two Groups

Variables a	Pre- Exercise			Post- Exercise		
	Exercise	Control	P value	Exercise	Control	P value [Table-fn fn1760]
**Age, y**	17.35 ± 1.070	16.90 ± 1.15	0.91	-	-	-
**Height, cm**	175.80 ± 7.30	171.20 ± 10.60	0.175	-	-	-
**Weight, kg**	87.26 ± 11.05	90.02 ± 10.50	0.49	83.90 ± 10.14	89.80 ± 9.70	0.116
**BF**	29.37 ± 1.85	29.17 ± 2.29	0.79	27.43 ± 1.30	29.11 ± 2.13	0.015^ a^
**BMI, kg/m^2^**	28.24 ± 2.56	28.31 ± 2.49	0.93	26.96 ± 2.37	28.85 ± 2.48	0.042^ a^
**VO2 max, mL/kg/min**	34.37 ± 2.50	33.55 ± 2.40	0.35	38.60 ± 2.16	33.70 ± 2.40	0.001^ a^
**ABCA1, pg/mg/p**	7.60 ± 3.36	5.74 ± 1.38	0.057	16.90 ± 7.10	6.67 ± 2.60	0.001^ a^
**APoA-I, µg/dL**	6.04 ± 1.68	6.98 ± 3.26	0.55	7.08 ± 2.50	6.62 ± 1.75	0.77
**H DL-c, mg/dL**	41.06 ± 4.80	46.10 ± 7.05	0.03^ a^	45.30 ± 7.40	44.5 ± 6.20	0.74
**L DL-c, mg/dL**	124.1 ± 27.7	141.5 ± 27.6	0.097	120.60 ± 22.03	150.20 ± 28.02	0.37
**TC, mg/dL**	200.30 ± 38.03	219.70 ± 38.60	0.177	183.80 ± 25.50	226.80 ± 34.90	0.001^ a^
**TG, mg/d**	172.57 ± 64.70	187.40 ± 118.10	0.68	145.40 ± 48.90	202.70 ± 69.90	0.017^ a^
**L DL-c/H DL-c**	3.05 ± 0.76	3.12 ± 0.65	0.79	2.81 ± 0.63	3.29 ± 0.70	0.026^a^
**TC/H DL-c**	4.91 ± 0.99	4.82 ± 0.86	0.78	4.13 ± 0.80	5.12 ± 0.77	0.002^ a^
**FG ,mg/dL**	97.90 ± 1.20	95.40 ± 10.07	0.58	97.70 ± 9.30	96.20 ± 9.60	0.66

^a^Data are presented as Mean ± SD

Abbreviation: ABCA1, ATP-Binding cassette transporter A1; Apo A-I,Apolipoprotein A-I; BF, percentage of body fat; BMI, body mass index; FG, fasting glucose; HDL-C, high-density lipoprotein cholesterol; LDL-c, low density lipoprotein cholesterol; TC, total cholesterol; TG, triglycerides

## 5. Discussion

The importance of regular exercise in preventing and treating chronic disease is generally accepted (25).The main findings of the present study were increase in lymphocyte ABCA1protein expression, plasma HDL-C, Apo-I and a reduction in plasma LDL-C concentrations following eight-week interval endurance rope training. Also there were significant changes in other lipid profiles and anthropometric indices. In the present study the increase of ABCA1 expression was in line with results of previously reported findings on different subjects with different training protocols, such as Butcher and et al.(17), Hoang , et al.(26) Ghanbari-Niaki et al. (18), and Rashidlamir & et al. (19). The mechanism(s) by which the exercise training can influence lymphocyte ABCA1 protein expression is (are) poorly understood. However, several possible mechanisms could be considered. It has been suggested that the modulating effect of fatty acids (FA) is mediated by peroxisome proliferator-activated receptors (PPARs) and it is also well known that PPAR is a nuclear receptor like liver X receptor (LXR) and retinoid X receptor (RXR) that regulates the expression of genes controlling lipid and glucose metabolism (18). Three PPAR isoforms (α, β/δ, γ) are widely expressed in metabolic tissues including the heart, liver, skeletal muscle, kidney and are also present in cells of the arterial wall including monocytes and macrophages (27, 28). The effect of exercise training on PPAR mRNA expression has been taken into consideration by several researchers (29, 30). Butcher et al. (17), have reported that 8 weeks of a low-intensity program lead to significant changes in human leukocyte liver X-receptor (LXR) and proliferator-activated receptor-α (PPAR-α) , they also found an increase in LXR and PPAR-α expression. They also suggested that importantly, ligand activation of PPAR-α also led to primary induction of LXR whose activation subsequently (after 4–8 wk exercise) triggered up-regulation of ABCA1 and ABCG1 and therefore increased RCT (17). Changes in plasma and tissue adiponectin concentrations and expression following an exercise training program was considered to be an effective factor to regulate ABCA1 expression (31, 32). It is shown that cAMP can also be enhanced ABCA1 gene transcription (25). In the current study there were increase in concentrations of ApoA-I and HDL-C and decrease in LDL-c value following IERT program but the changes weren’t significant, these results are consistent with the results of some studies (18, 33-36) contrast with some others (27, 35). It seems that increases in ABCA1 expression and preβHDL (as the first product of ApoA-I limitation), is the mechanism of increasing in Apo A-I (16, 37, 38). Also increase of ABCA1 expression and ApoA-I and some other factors involved in the process of cholesterol reverse transport (such as LCAT, CETP, PLTP and Scavenger receptor BI (SR-BI) are the main mechanisms of increasing HDL-c (16, 39). It seems that the lack of significant changes in ApoA-I and HDL-c and LDL-c is related to the volume and intensity of exercise. Studies have shown that effects of high volume and high intensity exercise on blood lipid profiles and apolipo proteins are more than those of the low or moderate exercise (34-36).Thus seems that the intensity and volume of exercise used in this study was not sufficient to cause significant changes in these three variables. Also the results of the current study showed a significant decrease in plasma levels of TG (that might be due to an accelerated TG removal by skeletal muscle which plays a major part of TG removal by peripheral tissues ) (18), TC, TC/HDL-c and LDL-c/HDL-c (as atherogenic indexes). These results are consistent with those of the other studies (34, 35). The present study also clearly showed that interval endurance rope training induced significant changes in VO_2_max, BMI and BF (%) in overweight and obese boy adolescents.In summary, this is the first report and direct evidence demonstrating that 8 weeks of interval endurance rope training program enhances lymphocyte ABCA1 expression which is accompanied with a little elevation in plasma HDL-C, ApoA-I concentrations. The present results also indicated that interval aerobic endurance exercise protocol could be taken into account as a modality of exercise for physical fitness improvement and weight control in all adolescents particularly overweight and obese boy adolescents.
